# Impact of Birth Preparedness and Complication Readiness Interventions on Birth with a Skilled Attendant: A Systematic Review

**DOI:** 10.1371/journal.pone.0143382

**Published:** 2015-11-23

**Authors:** Andrea Solnes Miltenburg, Yadira Roggeveen, Laura Shields, Marianne van Elteren, Jos van Roosmalen, Jelle Stekelenburg, Anayda Portela

**Affiliations:** 1 Department of Community Medicine, Institute of Health and Society, University of Oslo, Oslo, Norway; 2 Athena Institute for Research on Innovation and Communication in Health and Life sciences, Faculty of Earth and Life Sciences, VU University, Amsterdam, the Netherlands; 3 Department of International Mental Health, Netherlands Institute of Mental Health and Addiction, Utrecht, the Netherlands; 4 Department of Medical Humanities (EMGO) Institute for Health and Care Research VU, University Medical Center (VUmc), Amsterdam, the Netherlands; 5 Department of Obstetrics & Gynaecology, Leeuwarden Medical Centre, Leeuwarden, The Netherlands; 6 Department of Maternal, Newborn, Child, Adolescent Health, World Health Organization, Geneva, Switzerland; Medical University of Vienna, AUSTRIA

## Abstract

**Background:**

Increased preparedness for birth and complications is an essential part of antenatal care and has the potential to increase birth with a skilled attendant. We conducted a systematic review of studies to assess the effect of birth preparedness and complication readiness interventions on increasing birth with a skilled attendant.

**Methods:**

PubMed, Embase, CINAHL and grey literature were searched for studies from 2000 to 2012 using a broad range of search terms. Studies were included with diverse designs and intervention strategies that contained an element of birth preparedness and complication readiness. Data extracted included population, setting, study design, outcomes, intervention description, type of intervention strategy and funding sources. Quality of the studies was assessed. The studies varied in BP/CR interventions, design, use of control groups, data collection methods, and outcome measures. We therefore deemed meta-analysis was not appropriate and conducted a narrative synthesis of the findings.

**Results:**

Thirty-three references encompassing 20 different intervention programmes were included, of which one programmatic element was birth preparedness and complication readiness. Implementation strategies were diverse and included facility-, community-, or home-based services. Thirteen studies resulted in an increase in birth with a skilled attendant or facility birth. The majority of authors reported an increase in knowledge on birth preparedness and complication readiness.

**Conclusions:**

Birth Preparedness and Complication Readiness interventions can increase knowledge of preparations for birth and complications; however this does not always correspond to an increase in the use of a skilled attendant at birth.

## Background

The presence of a skilled attendant at birth (SBA) is promoted as a key strategy to prevent the leading causes of maternal and neonatal mortality and morbidity [1–3]. Despite a global increase in the number of births attended by SBAs, coverage in sub-Saharan Africa remains low [4]. This is the result of a combination of socio-economic, cultural and health system factors that cause delay in deciding to seek care (phase 1 delay), reaching maternal health care facilities (phase 2 delay) and receiving adequate care (phase 3 delay) [5]. Despite poor functioning health systems in low-and middle income countries [4,6,7] increased preparedness for birth and complications would allow women and their families to anticipate potential delays and ensure timely use of skilled care for birth and arrival at the appropriate facility for complications [8]. Implementation of birth preparedness and complication readiness (BP/CR) interventions that focus on individuals, families and communities are intended to reduce at least the first two delays [8]. It is equally important that health facilities and referral systems are prepared to deliver essential childbirth care and are able to manage complications, which would contribute to reduction of the third delay [9,10].

BP/CR is a process of planning for birth and anticipating actions to take in case of obstetric complications [10]. The concept of BP/CR emerged almost two decades ago and was later included by the World Health Organization (WHO) as an essential part of the antenatal care package [11,12]. According to WHO, BP/CR plans contain the following elements: desired place of birth; preferred birth attendant; location of the closest facility for birth and in case of complications; funds for any expenses; supplies and materials to bring to the facility; an identified labour and birth companion; an identified support person to look after other children at home; identified transport to a facility for birth or in case of complications; and identification of compatible blood donors if needed [13]. Acknowledging that not only women, but also families, communities, health care providers and policy makers need to be birth prepared, JHPIEGO developed a BP/CR matrix which conceptualizes multi-stakeholder preparedness (S1 Fig) [9,10,14].

A recent systematic review of randomized controlled trials (RCTs) showed that BP/CR strategies can reduce maternal and neonatal mortality [15]. However, seven out of the twelve included studies implemented BP/CR through action-learning cycles with women’s groups, a specific intervention and methodology which reported improvements to maternal and newborn health outcomes [16,17]. As the primary objective of BP/CR is to increase care seeking, mortality reduction also depends on accessibility and availability of services being provided. This makes the contributing effect of the BP/CR interventions on mortality less clear. In addition, change in mortality rates over time is difficult to assess and figures are often unreliable [18]. Therefore we set out to systematically review the literature, including qualitative studies, for the effect of BP/CR on increasing SBA [19].

The aim of this systematic review is to review the literature of BP/CR interventions and assess its effect on increasing SBA [19].

As there are several ways to implement and evaluate BP/CR interventions, we formulated the following key research questions to guide our review:

1. To what extent does BP/CR result in increasing skilled birth attendance?
2. What strategies are used to implement BP/CR?
3. What methodologies are used to measure the effectiveness of BP/CR?

Findings in this paper are also included in the WHO recommendations on heath promotion interventions for maternal and newborn health 2015 [20].

## Methods

In order to systematically synthesize the body of evidence, we followed the guidelines for systematic reviews of the Cochrane Handbook for Systematic Reviews of Interventions [21], the PRISMA statement [22] and the guidelines published by the National Health Service (NHS) Center for Reviews and Dissemination [23]. Details on the specific review methodology can be found in a prior publication (S1 File) [19]. The study protocol was registered at PROSPERO (no: CRD42012003124). Additional methodological considerations not mentioned in the study protocol or which were adjusted during the review process are described below.

### Literature search and selection process

We developed a search strategy (S2 File) for three electronic databases: PubMed, Embase and CINAHL. A wide range of search terms was used for high sensitivity as we anticipated that BP/CR terminology had only recently been used in publications. Originally we searched articles published between January 1987 and October 2012. However, this resulted in many irrelevant articles, in which concepts and interventions related to BP/CR were difficult to identify. We therefore excluded studies published before January 2000 and limited our search to the English language. We also manually searched grey literature and reviewed a database that included results of a systematic mapping of research on maternal health in low- and middle-income countries published from 2000 to 2012 [24,25]. The latter was limited to Arabic, English, French, Spanish, Japanese and Portuguese.

### Inclusion criteria

Studies were included if they were RCTs, quasi-experimental studies or comparative cohort studies which met the following criteria:

- Study population: pregnant women, women who recently gave birth, husbands of pregnant women or of women who recently gave birth, health care providers, traditional birth attendants (TBAs), all adults in the community (in low- and middle-income countries)
- Interventions: including BP/CR components, which could be facility-based, community-based or home-based implemented both as single intervention or as a package of interventions.
- Comparison: outcome reported must be compared with the outcome in any comparison group,
- Outcomes: birth with SBAs or facility births, maternal and neonatal mortality and morbidity, ANC with a skilled provider and knowledge of danger signs, implementation of BP/CR plan elements such as saving necessary funds, transport arrangements, etc

We excluded interventions that focused on increasing the quality of ANC provision and studies on facility training without the objective of increasing BP/CR. We also excluded descriptive studies on BP/CR, which did not evaluate any BP/CR intervention, but merely described barriers to BP/CR or use of SBAs.

### Study selection

**Fig 1:**
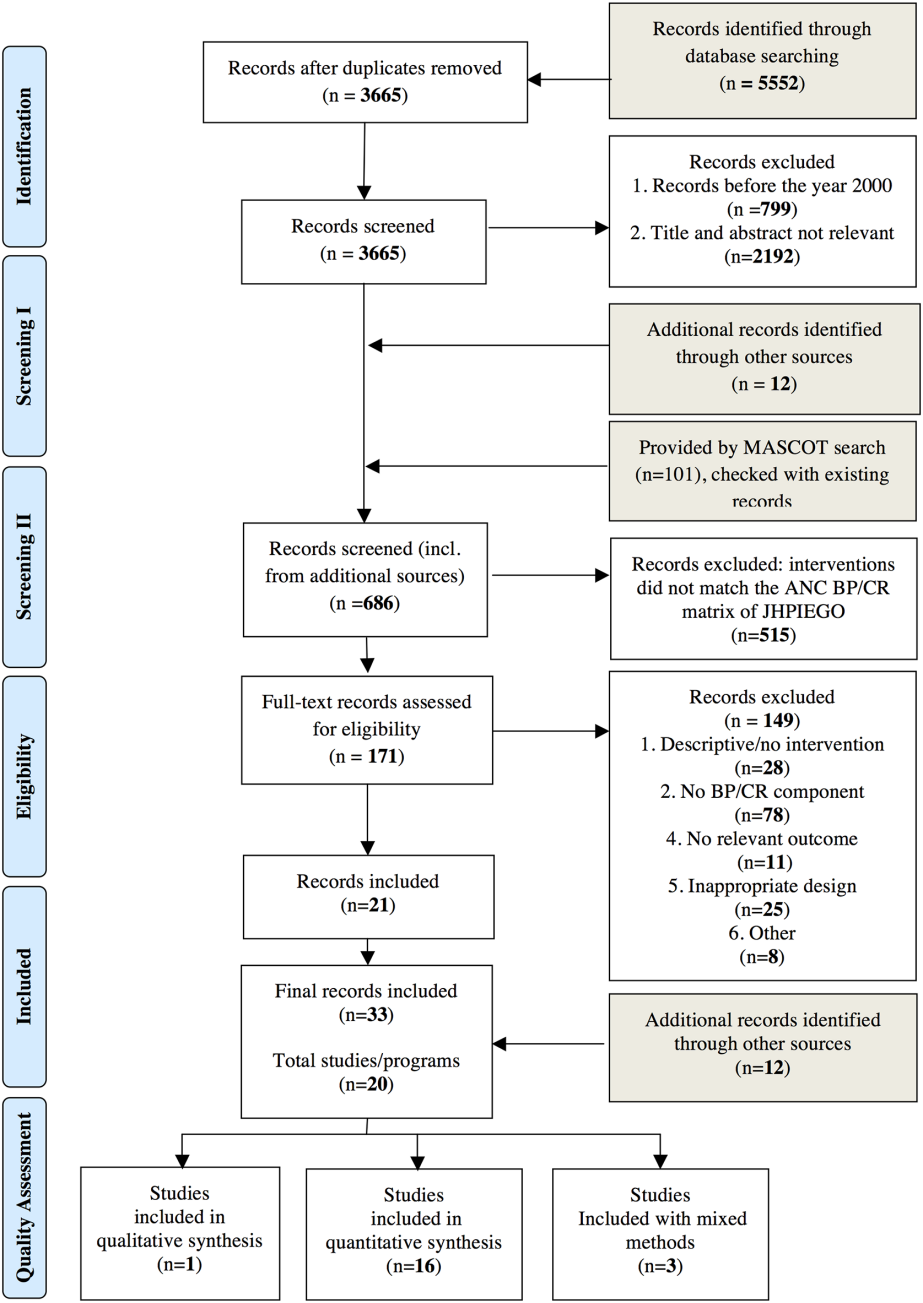
Prisma Flow Diagram [22].

Our search yielded 5552 records, of which 3665 remained after removal of duplicates (Fig 1). All abstracts and titles were searched and screened in duplicate and independently by ASM, YR and MvE. Of the 3665 records, 2991 were found not relevant or published before 2000. Remaining records (n = 674) and additional records identified (n = 12) were compared against JHPIEGO’s BP/CR matrix [10] to determine whether the study’s conceptualization aligned with the definition of BP/CR used in this review. Two reviewers reviewed the remaining 171 full text records independently for inclusion (ASM, YR, MvE or LS). Reference lists of the included records (n = 21) were hand searched for potentially relevant sources, yielding 9 additional records. Three additional studies were identified after presentation of our preliminary results at the WHO Technical Consultation on health promotion interventions for maternal and newborn health [26]. Disagreements on inclusion of studies were resolved by discussions with third parties (JS, JvR and AP). Included were 33 records covering 20 separate intervention programmes (e.g. some interventions or studies produced multiple articles).

### Data extraction

We tailored the NHS Center for Reviews and Dissemination data extraction table to fit our research questions [23]. Data extracted included setting, study design, outcomes, funding sources and intervention description. Data extraction by ASM was checked for accuracy and completeness by YR and LS. If additional data was needed, reports or data files were acquired by contacting authors and / or searching for study reports online.

### Quality assessment

ASM, YR, MvE and LS independently assessed the quality of the studies. Risk of bias for quantitative studies was assessed using the Effective Public Health Practice Project (EPHPP) quality assessment tool [27]. The quality of qualitative studies was assessed using the eight criteria developed by Walsh and Downe (2006) [28].

### Data analysis

Quantitative results were summarised in summary of evidence tables. The studies varied in BP/CR interventions, design, use of control groups, data collection methods, and outcome measures. We therefore deemed meta-analysis was not appropriate and conducted a narrative synthesis of the findings. [29].

## Results

All 20 programmes consisted of interventions wherein BP/CR comprised one element, either as a component (e.g. of ANC education), a sub-intervention (e.g. of a behavioural change strategy) or a primary intervention (e.g. administering a BP/CR package). See tables 1 and 2 for characteristics of included studies. The study designs include one RCT, three cluster RCTs, seven pre and post comparative studies with a control group, one pre and post study without control, seven one group before and after evaluations and one qualitative study. Three quantitative studies also had a qualitative component. Five of the 19 studies with a quantitative component received a moderate and 14 a weak rating. Assessment of four qualitative studies resulted in one moderate and three weak ratings (S1 Table).

**Table 1:** Study details for BP/CR interventions aiming to increase SBA for uncomplicated birth.

Study	Study Design	Description of intervention	Study population	No of participants	Programme name/ NGO
Brazier et al, 2009 [30]; Hounton et al, 2008 [31]; Graham et al, 2008[32]; Hounton et al, 2008 [33]; Newlands, 2008 [34]; Graham et al, 2008 [35] (Burkina Faso)	Pre-post with control	Behaviour change and community mobilization through participatory theatre and songs. Upgrading of health facilities and improving the referral system. Control district was also provided with facility upgrades but not the behavioural change component.	Women aged 15–49 who had experienced a pregnancy outcome between January 2002 and March 2006	Control n = 1311/1973, Intervention: n = 1178/1159	FCI; Skilled Birth Initiative
Family Care International Kenya, 2007[36]; Moor et al, 2002 [37]; Family Care International Kenya, 2003 [38] (Kenya)	Pre-post with control	Behaviour change campaign to increase use of skilled care before, during, and after childbirth making use of printed materials including BP messages through drama and meetings as well as supplied materials to HCW as well as facility upgrade and improving provider skills. Control district received only facility intervention.	Women who had given birth two years prior to the survey.	Baseline: 5,332 endline: 6,331 Women endline: 5,371 Husbands endline: 2,617	CHANGE PROJECT; Family Care International Skilled Care Initiative
Family Care International Tanzania, 2007 [39] (Tanzania)	Pre-post with control	Behaviour change communication and mobilization efforts to encourage health-seeking behaviours and build community support for the use of skilled care through participatory meetings at village level and theatre and performing arts. Improving the availability and quality of maternity care through strengthening physical infrastructure, improve provider skills. Control district received no intervention.	Women who had given birth two years prior to the survey.	Household: Baseline: 4,262 Endline: 4,804 Women endline: 5585, husbands endline: 3145	FCI; Skilled Birth Initiative Minister of Health and Social Welfare (MOHSW)
Sood et al, 2004 [40] (Nepal)	One group before after	A multi level behavioural change strategy with standardized safe motherhood messages incorporated into IEC/BCC materials such as posters, billboards etc. In addition mass media (Birth Preparedness Package) was developed to mobilize communities.	Women (pregnant), women (with live birth), husbands, family members and community leaders	Respondents were n = 1194 at baseline, n = 1208 endline	MNH Program; SUMATA initiative; JHU/ CCP; CEDPA; JHPIEGO; PATH; NHEICC of the Department of Health Services and the MoH
Sood et al, 2004 [41] (Indonesia)	Pre-post with control	Encourage and promote birth preparedness on each level directly targeting husbands, villages and communities through several (media) campaigns. In addition midwives received skills training both clinically as in communicating the basics of BP/CR to their clients during ANC.	Women who had a life birth in the past 15 months, the husband of every third woman, midwives and community influential’s	Baseline 2269 women, endline 1782 women	MNH Program; JHU/CCP; UNFPA; Ministry of Women Empowerment.
Fonseca-Becker et al, 2004 [42] (Guatemala)	One group before after	Service delivery improvements making use of Performance and Quality Improvement (PQI) and accreditation model and trained health care providers (through train the trainer approach) and Behaviour Change Interventions focused on organize communities to effectively respond to obstetric emergencies and creating demand for the improved services through the use of radio and printed materials	Women who recently delivered (<12 months).	Women: baseline n = 325endline n = 787, Men: baseline n = 512 endline n = 546	MNH Program; JHU/CCP; Guatemalan MoH
Moran et al, 2006 [43]; Baya et al, 2004 [44] (Burkina Faso)	One group before after	Community and facility based HCW and TBAs provided one-on-one counselling with pregnant women and families on key messages focused on birth preparedness and complication readiness using a flip-chart. These messages were reinforced through district-based radio messages and theatre plays. In addition facilities were upgraded and HCW were provided with additional training.	Pregnant women and women who recently deliveed (<12 months)	Recently delivered women (n = 180), pregnant women (n = 180)	MNH Program; PLAN; UNICEF Mwaganza Action
Mullany et al, 2007 [45] (Nepal)	RCT;	Intervention group consisted of couples and women alone who received health education (two sessions) provided by health educators. The curriculum covered a number of maternal health topics (Pregnancy care and birth preparedness, labour and delivery/postpartum period (12 and 17 topics within each session, respectively including complication readiness). Control group received no education, only a brief flyer designed to resemble and standardize the health education of normal care provided;	Pregnant women	Couples (n = 145), Women alone (n = 148), Control, (n = 149)	NA
Mushi et al, 2010 [46] (Tanzania)	One group before after	Training of safe motherhood promoters to educate and raise awareness on maternal health aspects for pregnant women, husbands and community members through home visits.	Female (pregnant, nursing mothers, and mothers) as well as male (husbands of the same) aged 18 years or above.	Baseline n = 238, Post-intervention n = 242	NA
McPherson et al, 2006 [47] (Nepal)	One group before after	CHWs through a Birth Preparedness Package use flipcharts and administer key chains to pregnant women, containing birth preparedness messages through monthly discussions in women’s groups. Facility-based CHWs counseled women who use facility-based services.	Mothers of live infants aged less than one year at the time of the survey.	Respondents per survey n = 300	Saving Newborn Lives (SNL); Save the Children-USA District Health Office, Family Health Division of Minister of Health and Population.
Turan et al, 2011 [48] (Eritrea)	Pre-post with control	Training of community members to become Maternal Health Volunteers (women and men) and lead participatory education sessions making use of materials developed. Training included BP/CR. Also skills training for health care providers.	Recently delivered women (<12 months)	Baseline n = 466, Endline n = 378	Eritrean MoH, UNFPA; Campaign to End Fistula, and the Stanford Eritrean Women’s Health Project.
Skinner et al, 2009 [49] (Cambodia)	Qualitative	Community development approach towards birth preparedness through dissemination and discussion of visual aids on danger signs and birth preparedness with families and communities	Midwives, village volunteers, TBAs, village chiefs, and mothers through	40 focus group discussions with a total of 327 participants.	NA
Hodgins et al, 2009 [50]; Valley Research Group 2007 (Nepal)	One group before after	Home based antenatal counseling on birth preparedness and complication readiness to pregnant women and family members making use of pictorial handouts by female community health volunteers.	Women who had delivered a live or stillborn child during the year before the interview date	1740 across two districts	Nepal Family Health Program (NFHP); Maternal Newborn Health Project
Sinha et al, 2008 [51] (India)	One group before after	The intervention sought to make maternal health a public concern through mobilizing communities. Home visits and through group meetings, family members were informed of how to take special care of pregnant women and help them access health care services. Awareness was raised among pregnant women about pregnancy-related care, antenatal care, institutional delivery and risk factors, and empowered them to access appropriate care.	All women who had delivered in the 12 months prior to the survey	Baseline survey n = 319; Endline survey n = 501	NA

**Table 2:** Study details for BP/CR interventions aiming to increase SBA in case of an emergency.

Study	Study Design	Description of intervention	Study population	No of participants	Program name/ NGO
Kumar et al, 2012 [52]; Kumar et al, 2008 [53] (India)	Cluster-RCT	Intervention package inc home visits, community meetings and folk-song meetings, maternal and newborn health stakeholder meetings, and meetings for community volunteers. Control clusters received standard care;	Women who delivered during study period	Intervention: 26 clusters, n = 2681, Control: 13 clusters n = 1129	Essential Newborn Care (ENC)
Darmstadt et al, 2010 [54] (Bangladesh)	Cluster-RCT	Birth and newborn care preparedness (BNCP) was promoted by trained CHWs through two antenatal home visits. CHWs conducted three additional postnatal visits to promote preventive newborn care practices and to identify and refer sick neonates. The control group received usual care services provided by the local and national government	Recently delivered women (within last 3 years before the survey).	Women of reproductive age: Intervention n = 9987, Control n = 11153.	NA
Midhet et al, 2010 [55] (Pakistan)	Cluster-RCT	Facilitators were trained to use booklets with pictures supported by a cassette with messages including birth preparedness messages. Intervention group consisted of a woman’s only and couples group. In addition TBA’s were trained for clean home delivery, owners of local vehicles were trained for referral. Healthcare providers in intervention and control arm received clinical training.	All ever-married women under 50 years of age; recently delivered woman (within 12 months before the survey).	Control: 1022, Women: 836, Couples: 703	NA
Ahluwalia et al, 2003[56]; Kaharuza, 2001[57]; Ahluwalia et al, 2010[58]; (Tanzania)	Pre-post no control	The VHWs were trained to educate pregnant women and their families on maternal and newborn health including to perform birth-planning counselling. In addition TBA’s were trained to recognize danger signs, facilities were upgraded and facility staff trained. A community surveillance system for pregnancies as set up.	Recently delivered woman (within 24 months before the survey)	Approximately 860 respondents for follow up survey.	Community Based Reproductive Health Project (CBRHP); CARE; CDC; MoH Tanzania
Hossain et al, 2006 [59]; Barbey et al, 2001[60] (Bangladesh)	Pre-post with control	TBA’s, fieldworkers, and village doctors were trained to disseminate BP messages incorporated into a variety of visual aids during home visits, group discussions at clinics, and village meetings. In addition development of community support systems and improvement of quality of care through a participatory approach and training of staff took place. Comparison district received facility upgrade but no community intervention, control district received no intervention.	Women, husbands, decision maker, newborn care takers and community agents	Intervention: n = 420, Comparison: n = 400, Control: n = 400	Dinjapur SafeMother Initiative; CARE; UNICEF; Government Bangladesh
Baqui et al. 2008[61] (India)	Pre-post with control	Home visits by auxiliary nurse midwives or aganwasi worker and change agents to provide counselling on preventive care, nutrition, and preparedness for childbirth, and health-care utilization for complications. Encourage families to call auxiliary nurse-midwife or trained traditional birth attendant to attend delivery. Postnatal visit by community-based worker as soon as possible after birth to provide counselling on breastfeeding, essential newborn care, maternal and newborn danger signs and health-care utilization.	Women who had had a live birth or stillbirth within the past 2 years.	Comparison: n = 6196/6014 Intervention n = 8756/7812	Integrated Nutrition and Health Programme (INHP) CARE-India, with the Indian government and local NGOs.

The studies were conducted in sub-Saharan Africa (n = 7), South East Asia (n = 12) and Central America (n = 1). The Maternal Neonatal Health (MNH) programme supported by the Johns Hopkins University Centre for Communication Programs (JHU/CCP) in Guatemala, Nepal, Indonesia and Burkina Faso and the Skilled Care Initiative of Family Care International in Burkina Faso, Kenya and Tanzania were multi-country programmes.

Results of studies are presented in Tables 3 and 4. We distinguished between BP/CR programs that aim to increase SBA for all births and those promoting SBA in case of complications. The latter took place in contexts with extremely low SBA and where the majority of births take place at home; consequently BP/CR messages are different and focused on care seeking for complications and the intervention also contributed to ensure safe birth practices at home.

**Table 3:** BP/CR interventions aiming to increase SBA for birth: Relevant outcomes and characteristics per study.

Study	Relevant improvement seen on primary outcome (SBA or FB)	Relevant improvement seen on secondary outcomes
Brazier et al, 2009 [30] (Burkina Faso)	Yes. SBA in the intervention district increased from 24% at baseline to 56% at endline (P<0.001, Chi^2^ test). This was similar for FB. In the control district a slight increase of birth with a SBA was seen from 32% to 36% (P<0.05, Breslow-Day Test of Homogeneity of Odds Ratios)	No: In the period 2002–2003, the pregnancy-related mortality risk was 5.8 per 1000 pregnancies in Diapaga, 3.7 per 1000 in the Ouargaye non SCI-intervention area and 4.9 per 1000 in the SCI-intervention area; with no significant differences between the areas. The pregnancy-related mortality risk declined over time in the SCI intervention area (34% reduction, P = 0.074), but the speed of decline was not significantly different from that seen in the non-SCI area (2% reduction, P = 0.933) or in Diapaga (10% reduction,P = 0.488). Hounton et al (2008)
Family Care International Kenya, 2007 [36] (Kenya)	No. In the intervention area SBA increased from 27% at baseline to 28% at endline (p-value not provided, authors report non significant). In the control area there was higher increase from 30% at baseline to 37% at endline (P<0.05)	Marginally: ANC visit at least 1 increased in the intervention group form 85% at baseline Intervention: baseline: 85% endline: 89% *P<0*.*01*. ANC visit >2 in the intervention group increased from 67% at baseline to 73% at endline*<0*.*01*. Similar changes were seen in control district. Birth preparedness counseling and information provided at ANC in the intervention group increased from 35% at baseline to 84% at endline *(p <* .*001)*, However an increase was also seen in the control area from baseline:29% to endline:81% *(p <* .*001)*
Family Care International Tanzania, 2007 [39] (Tanzania)	Yes. In the intervention area SBA increased from 48% at baseline to 54% at endline (p = 0.01) compared to the control area with a decline from 38% at baseline to 31% at endline (p-value not provided, authors report non significant)	Yes. Significant increase in exposed group (no significant change in unexposed area) for earlier ANC visit mean decreased from 7.0 to 6.1 months (p = 0.05) and ANC visit at least 1 increased from 88% at Baseline to 95% at endline *(p <* .*001)*. Improvements were seen in endline counseling in BP/CR (Increase from 18% to 35% in the intervention district p<0.001) and from 24%-32% in the control district (p<0.01)) and advice on where to give birth increased in the intervention district (44% to 57%, p < .001) but declined in control district (48% to 42%, p < .01).
Sood et al, 2004 [40] (Nepal)	No. Birth assisted by a doctor increased from 11.6% at baseline to 34.4% at endline. However, this was higher for the unexposed group (42%) compared to the exposed group (29.3%). Births attended by a nurse decreased from 0.8% at baseline to 0.0% at endline.	Partially. For knowledge there was a significant increase in exposed compared to unexposed for: vaginal bleeding as danger sign during pregnancy. An increase, but no significant difference between exposed/unexposed mentioned for severe post partum vaginal bleeding, high post partum fever, awareness of community schemes for transport and funds. A reduction was seen in Knowledge of prolonged labour as danger sign both in all groups (due to inconsistent terminology used). No effect was seen for retained placenta as danger sign. For practice, >4 ANC clinics attended in all groups, effect of intervention: *ns*. Arrangements for safe childbirth increased in all groups, effect of intervention: *ns*
Sood et al, 2004 [41] (Indonesia)	Marginal. Woman’s reported use of a SBA at birth decreased from 64.4% and baseline to 58.9% at endline. This decline was mainly due to lower reported use of health facility midwives (18% to 7.6%). There appeared to be an increase in birth with a SBA among the exposed group with significant difference between exposed and unexposed groups. Hospital birth did increase from 7.1% at baseline to 9.0% at endline (p<0.05) This was higher for the exposed group (11.4%) than the unexposed group (5.7%) (p<0.000)	Yes. Significantly higher awareness of vaginal bleeding as danger sign in pregnancy in all respondent categories, (e.g. women: 40.7% exposed group compared to 16.4% in unexposed group) and of vaginal bleeding during labour only in women (30,8% exposed to 12.3% unexposed), for postpartum bleeding significantly in all groups exposed compared to the unexposed. Significantly higher awareness of community assistance schemes in exposed group compared to unexposed. Emergency transport schemes were used more often by the unexposed. Knowledge of fever as danger sign decreased. For ANC visits there was no baseline data available for comparison
Fonseca-Becker et al, 2004 [42] (Guatemala)	Yes. FB increased from baseline (30.5%) to endline in the unexposed group to 31.2% and in the exposed group to 54.7%. P<0.01 between baseline and follow up P<0.01 between exposed and unexposed	Yes. Knowledge (of danger signs and community plans for transport and funds) increased significantly (between p<0.01 and p<0,05 for testing difference between exposed and not-exposed), except for fever as danger sign. Seeking care for ANC visits in second trimester increased significantly among those exposed (34.4%) compared to baseline (29.8%) p<0.05. Women who arranged finances for transport increased from baseline: 7.1% to endline (exp) 62.2% to (unexp) 26.2% p<0.01
Moran et al, 2006 [43] (Burkina Faso)	Yes. FB increased from 46.1% at baseline to 59.5% at endline. This was similar for SBA with an increase from 38.9% at baseline to 57.8% at endlline. For auxiliary midwives there was an increase from 15.6% at baseline to 41.7% at endline (p<0.05) higher for the exposed group (43.5%) versus the unexposed group (37.5%)	Yes. ANC visit >4 increased from 21.1% at baseline to 44.4% at endline (*p<0*.*05*). Similar improvements were seen for: timing of ANC >4 months (69.5% to 41.6% p<0.05); PNC 2x (20.5% to 50.6% p<0.01); For a birth plan made (pregnant woman reported): Preparations for transport increased from 37.3% at baseline to 51.1% at endline *(ns)*, financial preparations increased from 45.6% to 61.1% (p<0.05), Identified a Skilled provider increased from 66.7% to 70.6% (*ns*). For a birth plan made (recently delivered women reported): preparations for transport an increase from 2.8% at baseline to 46.1% at endline, financial preparations from 0.6% to 83.3% *(p<0*.*05*), discussed facility birth with husband increased from 18.5% to 52.2% (p<0.01) and the BP score >3 increased from 35.5% at baseline to 61% at endline.
Mullany et al, 2007 [45] (Nepal)	No. SBA in the different groups was 90.2% for the couples group, 89.6% for women only and 82.8% for the control group. Comparison for relative risk (RR) with 95% Confidence Interval was: Couples vs. Control: RR 1.09 (95% CI 0.99–1.20), Woman vs. Control: RR 1.08 (95% CI 0.98–1.19), Couples vs. Woman: RR 1,00 (95% CI 0.93–1.09) ns.	Marginally. Making > 3 birth preparations differed significantly for education of husbands and women when not living with the mother-in-law, in comparison to controls: 23% versus 4%. Comparison for relative risk (RR) with 95%CI was: RR 5.19 (95% CI 1.86–14.53) and significantly for women—not living with their mother-in-law- receiving education alone in comparison to controls: RR 4.44 (95% CI 1.56–12.69. Other group comparisons for birth preparedness *ns*. Women in couples group we more likely than women in control group to attend post-partum visit in facility with 61% versus 47%, RR 1.29 (95% CI 1.04–1.60) or woman-alone group 61% versus 49%, RR 1.25 (95% CI 1.01–1.54). There was no effect on ANC visits.
Mushi et al, 2010 [46] (Tanzania)	Yes. Compared to 34.1% at baseline, post-intervention SBA increased to 51.4% (p < 0.05). Similar trend for FB (Baseline 33.3%; Post-intervention 49.8%), p-value not provided	Marginally. ANC visits in primegravida <20 wk increased from 18.7% at baseline to 56.9% post-intervention *(p<0*.*01)*. Frequency of ANC visits and knowledge of danger signs was *ns*.
McPherson et al, 2006 [47] (Nepal)	No. SBA increased from 16% at baseline to 17% at endline (p = 0.55)	Yes. ANC 1+ visit increased from 60% at baseline to 84% at endline (*p = 0*.*000)*, ANC 2+ visit increased from 49% at baseline to 73% at endline (*p = 0*.*001)*. PNC within 6w increased from 17% to 34% at endline (*p = 0*.*02*), PNC within 1w from 11% to 25% at endline (*p = 0*.*01)*. The BP index increased from 33% at baseline to 54% at endline. Financial preparations from 45% to 72% (*p = 0*.*000)* and transportation preparations from 9% at baseline to 28% at endline (*p = 0*.*000)*
Turan et al, 2011 [48] (Eritrea)	Yes. FB in the intervention group increased from 3.2% at baseline to 46.8% at endline (OR 26.24 95% CI 11.42–60.27) compared to from 3.6% at baseline in the control group to 15.2% at endline (OR 4.80 95% CI 2.23–10.34) P = 0.003 (Breslow-Day Test of Homogeneity of Odds Ratios)	Yes. Significant for four or more ANC visits with increase from 18.5% at baseline to 79.5% at endline in the intervention group with Odds Ratio (OR) 17.09 (95% CI 9.85–29.66) and decrease in the control group from 53.2 at baseline to 47.4 at endline with OR 0.79 (95% CI 0.56–1.13) (*P <* .*001)*. The 1^st^ visit in 1^st^ trimester increased from 7.4% at baseline to 8.6% at endline with OR 6.72 (95% CI 3.34–13.52) compared to control group with slight increase from 8.5% at baseline to 8.8% at endline with OR 1.01 (95% CI 0.53–1.93) *(P =* .*001)*. For birth preparedness there was a significant difference in women who talked with a trained health worker (*P = 0*.*015)*, other indicators were *ns*. For knowledge of danger signs it was high in the control area at baseline, which did not, improving further, the intervention area showed significant improvements from baseline to endline.
Skinner et al, 2009 [49] (Cambodia)	Marginal. There was no baseline data collection in the intervention areas. Outcome data where extrapolated from existing data sources. Routine health facility data of the 10 facilities in the intervention area showed a 32% increase in the number of women giving birth with a midwife (2005 n = 271 and 2006 n = 357). The national average also increased in this period with 13%	No. There was no baseline data collection in the intervention areas. Antenatal care visits increased to 22% according to existing data of the facilities.
Hodgins et al, 2009 [50] (Nepal)	Marginal. Percentage of respondents who delivered in a health facility (among respondents with live birth) increased from 24.0% to 28.4% (OR 1.31 95% CI 1.10–1.57). In Banke the proportion rose markedly but in Jhapa, where the baseline rate was already high, there was little change.	Yes. Neonatal mortality decreased from 20/1000 (95% CI: 14 to 27) to 8/1000 (95% CI: 4 to 13) at endline. Adjusting for literacy and wealth differences between baseline and endline survey, as well as the cluster design, this yields an OR of 0.42 (95% CI: 0.24 to 0.72). Positive changes were seen in household practices for birth preparation. Setting aside money increased from 34.8% at baseline to 81.9% at endline (OR 9.78 6.93–13.80). Where 11.5% made arrangements for health facility delivery before birth at baseline, this increased to 19.9% at endline (OR2.10 1.62–2.71).
Sinha et al, 2008[51] (India)	Yes. Home birth decreased from 54.1% at baseline to 38.4% at endline (p<0.001). For facility birth, there was an increase from 7.9% to 16.0% in primary health facilities (p<0.001) and from 15.4% to 26.6% in the government hospital (p<0.001).	Yes. Care seeking for ANC increased for one antenatal check-up from 90.3%–95.8% (p<0.001), for more than three ANC visits from 87.2%-95.5% (p<0.001) and for ANC visit during 1^st^ trimester from 45.3%–54.9% (p<0.001). For birth preparedness an increase was seen in decision to deliver in an institution from 67.1% to78.6% (p<0.001), identification of hospital/facility for delivery from 40.2% to 65.3% (p<0.001), Identification and decision on transport from 28% to 52.1% (p<0.001), discussed birth related plans with close family members from 33.5% to 67.7% (p<0.001). Decrease was seen in identification of a birth attendant from 44.5% at baseline to 35.5% at endline.

**Table 4:** BP/CR interventions aiming to increase use of EmOC: Relevant outcomes and characteristics per study.

Study	Relevant improvement seen on primary outcome (SBA or FB)	Relevant improvement seen on secondary outcomes
Kumar et al, 2012[52] (India)	No. Births attended by a SBA increased from 14.3% to 26.9% in the intervention group compared to from 13.5% to 19.7% in the control group. Relative Risk: 1.37 95% Confidence Interval (0.92–2.03) P = 0.06	Yes, significant increase in intervention group compared to control in: 1). Recognition of danger signs in pregnancy: Swelling in hands and feet *(p*:*0*.*0001)*, Anemia *(p*:*0*.*0001)*, Fever *(p*:*0*.*05)*, Headache *(p*:*0*.*035*), Fits/convulsions *(p*:*0*.*01)* 2). Some, but not all, during labour; problems in placental expulsion (p>0.99), Obstructed labor (p>0.99), Breech presentation (p:0.56), Transverse/oblique presentation (p:0.0001), Excessive bleeding after delivery (p:0.0007), Prolonged labor (p:0.0001), Premature rupture of membrane (p:0.0014) 3). Birth preparedness practice: Preparation of room of confinement *(p*:*0*.*006)*, Prior identification of health facility *(p*:*0*.*0001)*, Prior identification of birth attendant *(p*:*0*.*048)*, Prior identification of delivery supervisor *(p*:*0*.*0006)* Prior arrangement of money (*p*:*0*.*018)* Prior arrangement of clean mattress *(p*:*0*.*0006)*. Unajusted and adjusted neonatal and perinatal mortality rates showed significant reductions in both intervention arms.
Darmstadt et al, 2010[54] (Bangladesh)	Yes. In the intervention area FB increased from 12.1% at baseline to 20.2% at endline In the control area there was an increase from 12.5 at baseline to 16.5% at endline (P<0,05)	Marginally. Increased knowledge in intervention compared to control was seen for: danger signs antenatal with an increase from 1.0% at baseline to 2.2% at endline in the intervention group to Increase in the comparison group from 1.1% at baseline to 2.9% at endline (P<0.05). For danger signs during labor/delivery the intervention group increased to 1.9% ar endline from 1.1% at baseline. Comparison showed an increase from 1.2% at baseline to 3.4% at endline *(P<0*.*05)*. Danger signs post partum increased in intervention from nil at baseline to 2.0% at endline. Comparison from 1.0% at baseline to 2.5% at endline *(P<0*.*05)*. Neonatal danger signs from 2.3% at baseline in intervention group to 2.4% at endline. Comparison from 2.3% at baseline to 2.8% at endline *(p<0*.*05)*. Increase in practice was seen for >1 ANC visit with skilled provider in intervention area: increase from 47.7% at baseline to 68.8% at endline, and in control area a sligh increase from 47.8% at baseline to 49.1% at endline *(P<0*.*05)*. No significant differences ìn NMR estimates over time and study arm.
Midhet et al, 2010[55] (Pakistan)	Yes. Both intervention groups: couples (4.1%) and women only (3.9%) showed higher percentage of FB in the District Hospital than the control group (2.9%) (p<0.05). AOR for women’s only group 1.3 (95% CI: 0.7–2.5) and for couples 1.3 (95% CI: 0.6–2.7).	Yes. Perinatal mortality and early neonatal mortality were significantly lower in intervention group. Perinatal mortality was 95.6% in control, 48.7% in women’s only and 67.2% in couples group (P<0.05). Early neonatal mortality: was 39.1% in the control 24.3% in the women only and 17.7% in the couples group (P<0.05). Neonatal mortality was 48.0% in the control group, 32.4% in the women only and 30.5% in the couples group (ns) Also significant more women had a Prenatal check up in 1^st^ /2nd trimester both in women’s (31.6%) and in couples group (38.2) compared to the control group (12.4%)
Ahluwalia et al, 2003[56] (Tanzania)	No. Delivery assisted by a health provider decreased from 56% in 1997 at the start of the study to 49% in 2001.	Yes. Household with a pregnant woman who had a birth plan in place increased from 0 at baseline to 48% at endline. Pregnant women who were able to identify 2 or more danger signs during pregnancy and delivery increased from 10% to 56% at endline. Obstetric complications attended at the district hospital increased from 4% to 15% at endline. A total of 44 of 52 communities had descriptions of action plans for transporting people with health emergencies to health facilities, and 12 (23%) had a specific system in place to implement the transport system (e.g. had collected funds)
Hossain et al, 2006[59], (Bangladesh)	Yes. The intervention area had an 8.1% increase of FB p<0.01 95%CI 7.2–9.0. (From 2.4% pre-intervention to 10.5% post intervention). Both control and comparison area had higher pre-intervention FB but significant less increase post-intervention: 0.5% in the control area (from 4.5% to 5.0%) and 5.3% in the comparison area (from 7.2% to 12.5%)	Yes. The intervention area had a 23.8% increase of met need for EmOC (16.0%–39.8%) compared to the comparison area which had a 13.0% increase (12.5%–25.5%) and the control area with a 1% increase (11.1%-12.1%). Knowledge of >3 danger signs was higher in the intervention area (45%) compared with 4% in comparison and 6% in control. Knowledge of birth planning messages was also higher in the intervention area. For more than 3 messages 20% compared to 2% and 1% in comparison and control. For 1 or 2 messages 45% compared to 26% and 19% in comparison and control.
Baqui et al. 2008[61], (India)	Yes. Delivered in a health facility or at home with a skilled birth attendant increased from 16.3% at baseline to 22.5% at endline in the intervention district. Similar increase was seen in the control district from 17.5% to 21.8% (p <0.009, *P*-value for difference-in-difference test adjusted for age, education, parity, religion and standard-of-living score)	Yes. Improvements were seen in behavioral change towards increase in >1 and >3 ANC visits With an increase in >1 ANC visits from 16.6% at baseline to 35.5% at endline in the intervention site compared to 24.5% at baseline to 27.5% at endline in the control district (p<0.001). Similar changes were seen for birth planning. In the intervention site saving money for childbirth increased from 14.8% to 50.4% compared to 12.2%–29.9% in the control site (p<0.001). No effect was seen on neonatal mortality rate.

### Effect on birth with a skilled attendant

Across multi-country programmes, i.e. Skilled Care Initiative and the MNH programme, results varied. The Skilled Care Initiative found increases in SBA in Burkina Faso and Tanzania, but not in Kenya [30,36,39]. Exposure to BP/CR interventions in Tanzania correlated with increased likelihood of seeking skilled care during childbirth. Of respondents exposed to ANC counselling on BP/CR 74% sought skilled care versus 64% of those unexposed (p<0.05) [39].

The MNH programme resulted in an increase in facility births or birth with SBAs in Burkina Faso and Guatemala [42,43]. No improvements were found in Nepal and Indonesia [40,41]. In Burkina Faso improvements were mainly due to an increase in births assisted by auxiliary midwives from baseline to endline (15.6% to 41.7%, p<0.05), which was higher for the exposed group. All authors of the MNH programmes reported an increase in knowledge of BP/CR and increase in BP/CR actions, however, this did not necessarily increase seeking skilled care [43].

In Tanzania, an intervention package, comprising training of Safe Motherhood Promotors and education on the importance of a birth preparedness plan through home visits, showed an increase of 51.4% in SBA post-intervention compared to 34.1% at baseline (P<0.05) [46]. Turan et al. (2011) trained community members (women and men) as Maternal Health Volunteers to lead participatory education sessions (including BP/CR) using visual aids. Facility births in the intervention group increased (3.2% to 46.8%, (OR 26.2, 95% CI 11.4–60.3), while the facility births in the control group increased from 4% to 15%. In India, a birth preparedness intervention geared towards families and communities resulted in an increase in births at primary care facilities (p<0.001) and government hospitals (p<0.001) [51].

Of the six studies of interventions aiming to increase access to care for complications, three resulted in increased facility births [54,59,55]. Hossain et al (2006) implemented a multi-stakeholder intervention consisting of facility-based interventions (facility upgrades and improvements in quality of care) and community interventions addressing birth planning and community mobilization to ensure timely recognition and referral of obstetric emergencies. The intervention site received all interventions, the comparison site only a facility upgrade and the control site received none. The intervention area showed an 8.1% increase in facility births (p<0.01 95% CI 7.2–9.0); however, both the control and comparison area had a higher pre-intervention facility birth rate. Darmstadt et al (2010) found a significant increase of facility births in the intervention area (from 12.1% at baseline to 20.2% at endline) compared to the control area (increase from 12.5% at baseline to 16.5% at endline—P<0,05)[54].

Most authors reported a statistically significant improvement in knowledge on BP/CR (Table 4). Mullany et al (2007) showed that couple counselling significantly improved knowledge compared to individual counselling and they suggested that immediate conversations between spouses might enhance knowledge retention [45]. Knowledge acquired was not always consistently related to the intervention as was shown by Sood et al (2004), who found that knowledge of danger signs was higher in the control group [40].

ANC attendance was not evaluated in all BP/CR studies. Study results varied from a significant increase in ANC attendance [36,39,42,47,48,54,43], earlier booking dates [46,48] to not any effect [40,45]. Different outcome measures and cut-off points for frequency or timing of ANC visits were used.

Of the five studies reporting on neonatal mortality, Kumar et al (2012) report significantly lower neonatal mortality in the BP intervention group [52] and Hodgins et al (2009) showed fewer neonatal deaths over time [50]. No significant difference was found in other studies.

### Strategies to implement BP/CR

After reviewing the studies on strategies used for BP/CR implementation, we grouped strategies into five categories. Some interventions used multiple strategies: education through home visits by volunteers or community health workers [43,46,49–52,54,56,59,61], BP/CR messages integrated into ANC education at facility level [41,42,45,43], visual aids with BP/CR messages such as booklets or flipcharts [36,39–43,47,49,50,55,59], participatory community mobilization activities including drama, songs and dance [30,36,39–41,43,46,48,49,55,59] and media campaigns (e.g. radio spots, jingles or television dramas). The majority of studies were published between 2004 and 2012 suggesting increased interest in BP/CR and using the label of BP/CR after introduction by JHPIEGO.

### Methodologies to measure effectiveness

Definitions of BP/CR varied from identifying a place of birth and preferred SBA, to preparing funds for complications, to arranging for (emergency) transport and knowledge of danger signs. Focus was either solely on the mother, or on both the mother and newborn. Across studies, household surveys were most frequently employed to evaluate programme effectiveness. Although interventions targeted different study populations (women, husbands and mothers in law), authors almost exclusively evaluated women’s behaviour as primary outcome [62–68]. The study population was heterogeneous across studies, ranging from pregnant women who gave birth during the study period [45,52] to women who had ‘recently’ given birth. A number of multi-country programmes had outcome measures at family or health worker level. Some authors measured facility birth as an indicator for SBA. Methods to assess if women were birth prepared and complication ready differed greatly across studies, due to varying scales and index criteria used.

## Discussion

Heterogeneity of study designs and BP/CR interventions, and lack of high quality evidence prevents making a pooled analysis. Although BP/CR interventions can increase knowledge of danger signs and preparations for birth and complications, this did not always correspond to an increased use in SBA at birth. Where an increase in SBA or facility birth was reported, BP/CR interventions were primarily part of a package of multiple interventions and involved multiple stakeholders, making it difficult to attribute the effect to the BP/CR component alone.

Interventions where BP/CR was a primary component can be better assessed in terms of causality, but what these results mean in a complex reality is unclear [45,46,69]. Many variables influence both programme interventions and outcomes such as female education and policy changes [48,70]. Active involvement of policy makers in BP/CR interventions facilitated implementation at the national level in some countries [30,40–43]. We will analyse this further in a separate publication [70]. Increased use of SBA in BP/CR programmes within a package of interventions could be due to facility or infrastructure improvements, community-based behavioural change interventions, other factors, or due to interactions between all [30].

Although the JHPIEGO BP/CR matrix includes preparedness of facilities and health providers, BP/CR studies rarely focus on the supply side of skilled care [10]. Ensuring that services are equipped to meet the increased demand likely to be generated by BP/CR interventions is crucial. Advising community members to prepare for facility birth, while health services or health providers are not birth prepared or complication ready, or while local health systems are not ready for an increased caseload, can lead to an increase of in-facility complications or maltreatment. Consequently, negative in-facility experiences can increase delay in care seeking [5] and should be avoided as much as possible. Also, negatively contributing factors at ANC need to be addressed for proper BP/CR counselling such as insufficient human resources and time constraints [69,71,72].

Although most studies report increased knowledge of BP/CR, not all clarify whether this resulted in plans or actions. Knowledge alone does not equate to an increase in care-seeking behaviour, especially for maternity care services, often due to financial, structural, geographical or cultural factors [73,74]. Studies that focussed on ‘knowledge on danger signs’ and ‘preparing transport and funds in the event of an emergency’ predominantly aimed to increase access to Emergency Obstetric Care (EmOC) in case of complications. However, most births start uncomplicated and risk identification is unreliable [75]. Delays in reaching skilled care are partially caused by delayed recognition of signs and symptoms of labour onset [76,77]. We argue that BP/CR programmes should follow Safe Motherhood programmes in their shift towards the promotion of skilled care for all births and include education on the signs of uncomplicated labour to ensure timely preparations [78].

The strength of this review lies in its broad literature base, including published and unpublished studies (e.g. reports from NGOs). Although we limited our initial search to English language, the systematic mapping of maternal health research did not have this limitation. It is likely that we included all relevant studies by crosschecking our search results with this broad database and by being open for inclusion of additional articles at the WHO Technical Consultation [26]. Facility birth in many studies was used as an indicator for birth with SBAs, this must be interpreted with caution as many facilities may lack the availability or presence of SBAs [35]. Similarly only four studies presented their definition of an SBA and it is unclear if in the other studies a SBA was defined according to our definition. Despite wide spread promotion of BP/CR through the JHPIEGO and WHO publications, definitions and indicators of BP/CR varied greatly across studies, therefore comparing studies was challenging which also prevented the possibility of conducting a meta-analysis.

## Conclusion

Although BP/CR in theory is compelling as a strategy to increase birth with a SBA, robust evidence of the effect of BP/CR in itself on increasing birth with a skilled attendant remains limited. This review does suggest that BP/CR interventions in combination with other interventions have the potential to increase use of SBAs and to increase timely use of facility care for birth and obstetric and newborn complications. We argue that BP/CR interventions seem as strong as the weakest link in the continuum of maternal care pathway.

### Recommendations

Clarification of definitions of BP/CR is needed to guide future programme implementation and evaluation. Expert meetings and internationally-agreed upon definitions and indicators for BP/CR could help. However as specific actions and messages required to prepare for birth and complications are highly context specific, it seems undesirable to aim for uniformity. Creating a flexible BP/CR definition that allows local adaptation is a step forward. Collaboration between target groups is a crucial step, and requires further study. An excellent way to locally define and implement BP/CR programmes would be to develop and study local BP/CR pathways collaboratively with target groups from community to policy level. The JHPIEGO matrix is a helpful tool to start this process. Study of this process and outcomes, should include mixed methods by transdisciplinary research teams [79].

## Supporting Information

S1 Fig Birth Preparedness and Complication Readiness Matrix. (TIFF)

S1 File Protocol for Systematic Review. (TIFF)

S2 File Search Strategy. (TIFF)

S3 File Prisma Checklist BPCR. (TIFF)

S1 Table Quality Assessment. (TIFF)
