# Supplementary figures and images for: Impact of Birth Preparedness and Complication Readiness Interventions on Birth with a Skilled Attendant: A Systematic Review
Source: PLoS One. 2015 Nov 23;10(11):e0143382. doi: 10.1371/journal.pone.0143382 (PMC4658103; doi:10.1371/journal.pone.0143382)

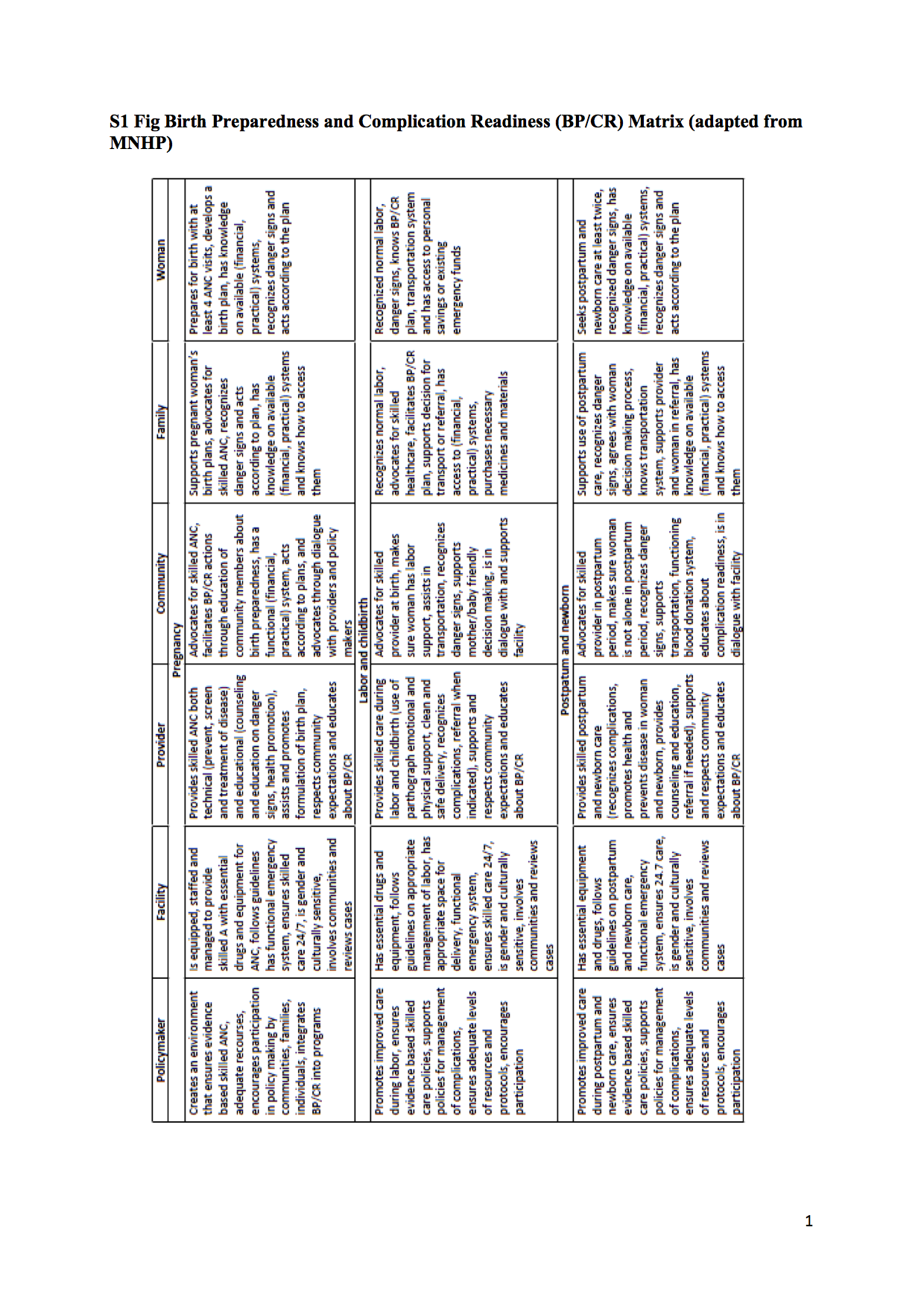

Supplement: S1 Fig — (TIFF) [file pone.0143382.s001.tiff]

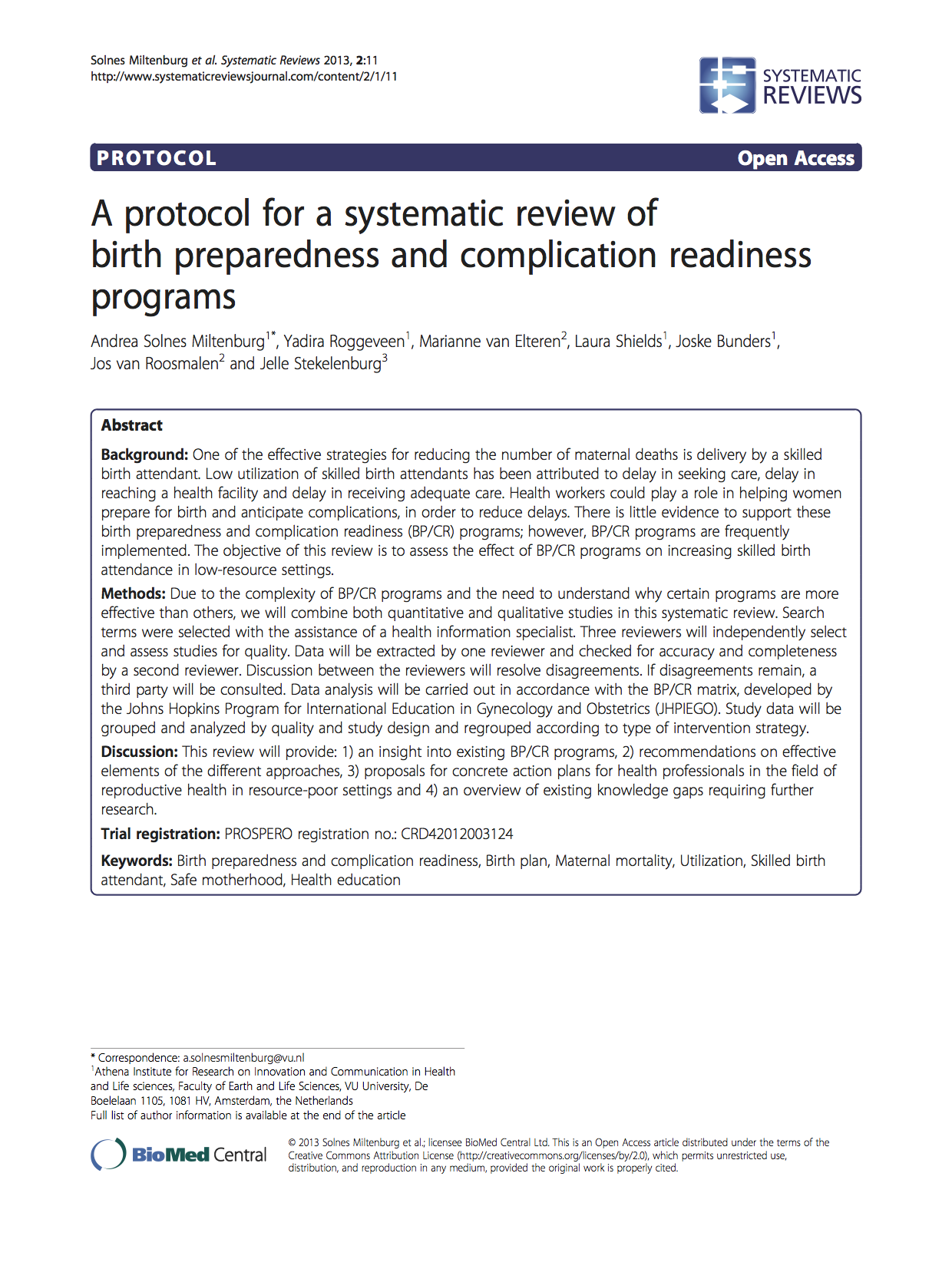

Supplement: S1 File — (TIFF) [file pone.0143382.s002.tiff]

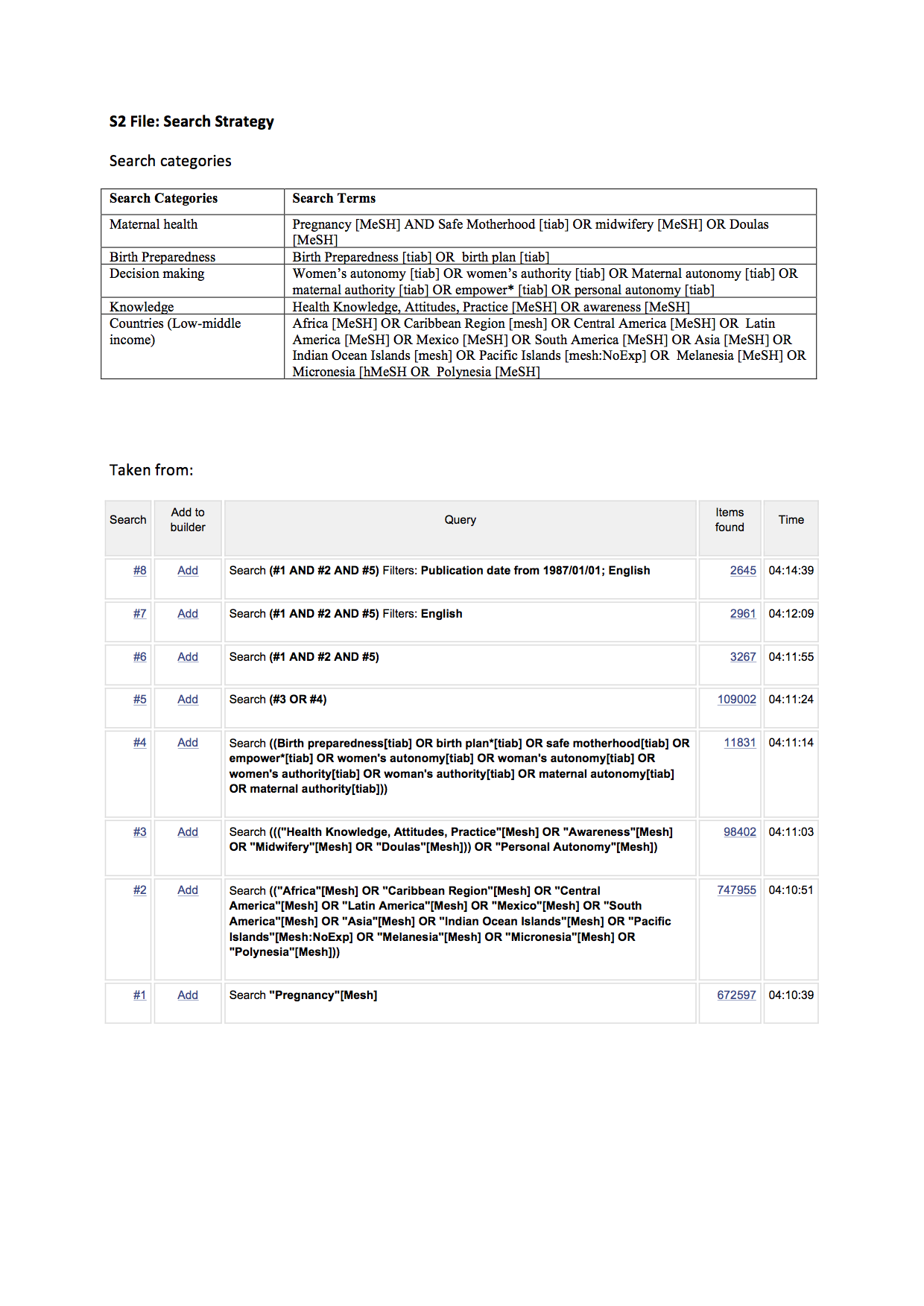

Supplement: S2 File — (TIFF) [file pone.0143382.s003.tiff]

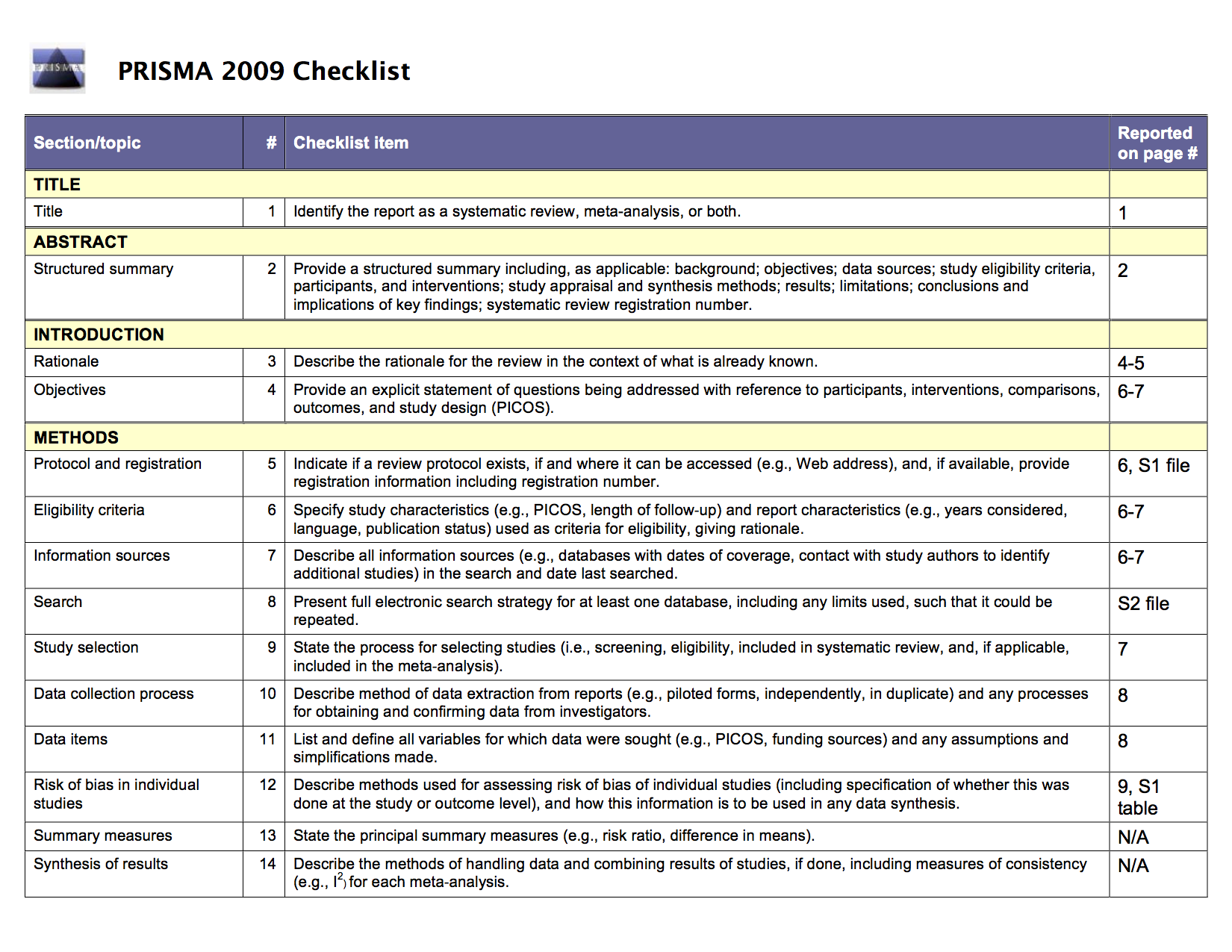

Supplement: S3 File — (TIFF) [file pone.0143382.s004.tiff]

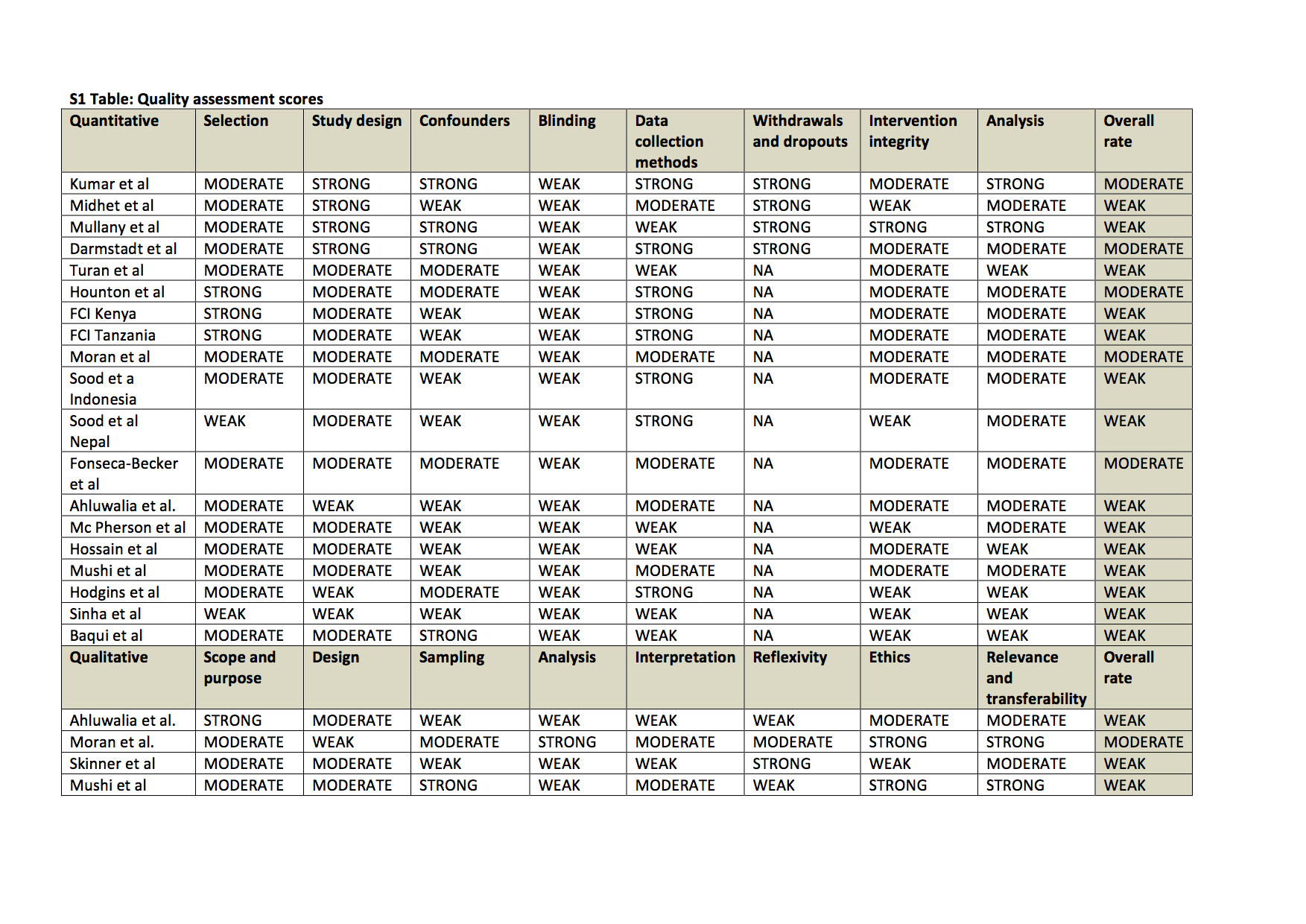

Supplement: S1 Table — (TIFF) [file pone.0143382.s005.tiff]
